# Study on settlement prediction of soft ground considering multiple feature parameters based on ISSA-RF model

**DOI:** 10.1038/s41598-024-55236-w

**Published:** 2024-03-01

**Authors:** Changshuai Sun, Tianwen Yu, Min Li, Huanwei Wei, Fang Tan

**Affiliations:** 1 Shandong Electric Power Engineering Consulting Institute Corp., Ltd, Jinan, 250013 China; 2 School of Civil Engineering, Shandong Jianzhu University, Jinan, 250101 China; 3 Approval Service Section III, Administrative Approval service Bureau of Chengyang District, Qingdao, 266109 China; 4 Key Laboratory of Building Structural Retrofitting and Underground Space Engineering (Shandong Jianzhu University), Ministry of Education, Jinan, 250101 China

**Keywords:** Civil engineering, Computational science

## Abstract

By collecting a large amount of data from various preloading engineering projects, a settlement prediction database was established including up to 15 feature parameters, such as final measured time, magnitude of surcharge loading, porosity ratio, internal friction angle, and others. Furthermore, a settlement prediction model of soft foundation based on random forest (RF) model was also developed. To enhance the accuracy of settlement prediction, the improved sparrow search algorithm (ISSA), which incorporates several enhancements such as the use of Logistic-tent chaotic mapping, adaptive nonlinear inertia-decreasing weight parameters, and Levy flight strategy, was proposed to optimize the hyperparameters of the RF model. The optimization results of various algorithms on benchmark functions revealed that the ISSA algorithm excelled in terms of accuracy and stability when compared to conventional algorithms such as particle swarm optimization and butterfly optimization. The ISSA-RF settlement prediction model was subsequently constructed and applied to practical projects. The results demonstrated that the ISSA-RF model exhibited superior prediction accuracy and applicability compared to the RF model. It can therefore provide valuable guidance for the planning and implementation of preloading engineering projects.

## Introduction

In coastal regions, dredged-sand disposal is commonly utilized to expand the land area through the deposition of sediments. However, the newly created land typically consists of silty soil, which exhibits inadequate bearing capacity and needs to be treated by preloading. To determine the reasonable unloading time, it is necessary to make accurate predictions of the settlement before and after unloading according to the development law of foundation settlement during surcharge. At present, the widely used settlement prediction methods are mainly divided into traditional methods, numerical simulation methods and artificial intelligence methods.

The traditional method for settlement prediction relies primarily in-site measured data. This approach involves fitting the trend of settlement development to predict future settlements. Pan et al.^1^ introduced the utilization of curve fitting as a method for predicting settlement in soft ground, and Huang et al.^2^ proposed settlement prediction based on the Asaoka method. However, due to the rapid development of settlement in the early stage of the surcharge preloading project and the slow development of the later settlement, it is challenging to select an appropriate fitting curve solely based on early settlement trends for accurate predictions.

The numerical simulation method employs finite element software for modeling analysis to predict settlement. Xu et al.^3^ utilized the Plaxis numerical software to accurately predict the post-construction settlement of the soft foundation at the airport, while Muething et al.^4^ encountered significant discrepancies between their predicted results and field measurements when using Plaxis 2D and Plaxis 3D to predict the settlement of the soft clay foundation. The primary factor contributing to this is the significant reliance of numerical simulation predictions on the model and soil parameters provided by the institution. However, in practical projects, variations such as soil disturbance and other factors can lead to differences between the original soil parameters and those obtained through testing. These parameter deviations can significantly influence the prediction results, resulting in disparities between the numerical simulation predictions and actual outcomes. It is essential to modify the soil parameters through parameter inversion^5^, as the modified soil parameters can improve the accuracy of the model. Nevertheless, this approach also elevates the intricacies and complexity of the prediction process.

With the rapid development of artificial intelligence, intelligent prediction methods based on machine learning have begun to be applied in all walks of life. Sihag et al.^6^ used random forest and other machine learning algorithms to predict travel times in heterogeneous and disordered traffic conditions in India. Samui et al.^7^ used machine learning algorithm to study the impact of foundation settlement on structural durability. Ma^8^ combined with factor analysis and BP neural network to improve the settlement prediction model, achieving a prediction error of less than 10%. However, during the early stage of application, artificial intelligence settlement prediction often only considers the correlation between settlement and time, disregarding the impact of site construction characteristics and soil parameters on settlement. To solve this issue, scholars have begun establishing settlement prediction databases based on actual engineering monitoring data^9,10^. Yet the construction of settlement prediction databases has mainly focused on shield excavation engineering, and is less used in the treatment engineering of soft foundation.

When utilizing a machine learning model for settlement prediction, the performance of the model is influenced by its hyperparameter values. To improve the predictive capabilities of machine learning models, intelligent optimization algorithms are often employed to identify the optimal hyperparameters^11^. However, traditional intelligent optimization algorithm exhibit limitations such as insufficient population diversity, slow convergence speed, and susceptibility to falling into local optimization. Therefore, there is a need to improve traditional intelligent optimization algorithms to enhance their optimization performance^12–14^.

To address the aforementioned challenges, this paper first collects a substantial amount of actual preloading projects, and sorts out the settlement data obtained during the preloading period. Subsequently, a settlement prediction database is established, which contains on-site construction information such as preloading methods, soil parameters, and other characteristics. Then, multiple strategies are employed to enhance the efficiency of the sparrow search algorithm, and the corresponding settlement prediction model is proposed. Finally, the performance of the proposed model is evaluated based on the data in the established database by comparing with the existing model, and the implementation of the models in the actual project is also evaluated.

## Construction database

### Database collection

To enhance the generalizability of the proposed method and improve prediction accuracy, preloading projects should be collected as many as possible. Similarly, the database should contain as much construction information as possible. This will enable the algorithm to capture more patterns and trends of settlement, leading to more accurate predictions. Therefore, the numerous on-site preloading projects were collected in this study from the published researches, and information such as reinforcement treatment method, drainage method, drainage layout mode and measured settlement curve was obtained. The settlement prediction database contains 105 measured points and 893 sets of settlement data. The datasets used in this study are presented in Table 1, and the star symbol indicates that this project encompasses the unloading stage.

**Table 1:** Database constructed for predicting settlement.

Projects	Treatment methods	Projects	Treatment methods
Yanez et al.^15^	Vacuum combined with surcharge preloading*	Liu et al.^16^	Vacuum preloading
Yao et al.^17^	Vacuum combined with surcharge preloading	Cai et al.^18^	Vacuum preloading
Gouw^19^	Vacuum preloading*	Ding et al.^20^	Vacuum combined with surcharge preloading
Jun et al.^21^	Vacuum combined with surcharge preloading*	Ling et al.^22^	Vacuum preloading
Karunawardena^23^	Vacuum combined with surcharge preloading	Long et al.^24^	Vacuum combined with surcharge preloading*
Hoang et al.^25^	Vacuum combined with surcharge preloading	Geng et al.^26^	Vacuum combined with surcharge preloading
Indraratna et al.^27^	surcharge preloading	Zhong et al.^28^	Surcharge preloading
Hansbo et al.^29^	Surcharge preloading*	Doyle et al.^30^	Vacuum combined with surcharge preloading
Sun et al.^31^	Vacuum preloading*	Bergado et al.^32^	Surcharge preloading
Quang et al.^33^	Surcharge preloading	Yan et al.^34^	Vacuum combined with surcharge preloading
Chen et al.^35^	Surcharge preloading	Yang et al.^36^	Surcharge preloading
Li et al.^37^	Surcharge preloading	Ding et al.^38^	Surcharge preloading
Tan et al.^39^	Surcharge preloading*	Xie et al.^40^	Vacuum combined with surcharge preloading
Wang et al.^41^	Surcharge preloading	Jia et al.^42^	Surcharge preloading
Zhang et al.^43^	Surcharge preloading	Mu et al.^44^	Vacuum combined with surcharge preloading
Liu et al.^45^	Surcharge preloading	Liu et al.^46^	Vacuum preloading
Yu et al.^47^	Surcharge preloading	Li et al.^48^	Surcharge preloading

The settlement prediction database encompasses various methods for treating soft foundation, such as surcharge preloading, vacuum preloading, and vacuum combined surcharge preloading. Notably, vacuum combined surcharge precompression accounts for 45% of the database. The drainage methods include plastic drainage board, sand well, and precipitation well, with plastic drainage board comprising 75.3% of the data. The drainage layouts include triangle, square, and plum blossom shapes, with the square and triangular layouts being the most common, accounting for 56.2% and 41.9%, respectively. The plum blossom shape comprises the smallest proportion, at only 1.7%. Additionally, the database includes projects that involve the unloading stage, representing 36.19% of the data.

### Data preprocessing

To more accurately reflect the changes occurring in the soil layers during the process of database collection, the soil layer was divided into four segments of equal length based on the depth of the drainage facility. For every segment *j,* the weighted average of the compression modulus $\overline{E}_{sj}$, internal friction angle $\overline{\varphi }_{j}$, and porosity ratio $\overline{e}_{j}$ was calculated using the following equations:

1 $$\overline{E}_{sj} = \frac{{\sum {E_{si} h_{i} } }}{{\sum {h_{i} } }}$$

2 $$\overline{\varphi }_{j} = \frac{{\sum {\phi_{i} h_{i} } }}{{\sum {h_{i} } }}$$

3 $$\overline{e}_{j} = \frac{{\sum {e_{i} h_{i} } }}{{\sum {h_{i} } }}$$

where *h*_*i*_ is the thickness of the *i*th layer of soil in segment *j* (*j* = 1, 2, 3 and 4), and *E*_*si*_, $\phi_{i}$, and $e_{i}$ represent the survey results of *i*th layer of soil. After preprocessing the database, the input and output parameters of the initial settlement prediction model can be obtained as shown in Table 2. This data processing approach allowed for a more comprehensive representation of the mechanical properties of the soil and their variations within each segment.

**Table 2:** Parameters of settlement prediction model.

Classification	Parameters
Input parameter	(1) *SM*: Surcharge method
	(2) *DM*: Drainage method
	(3) *LM*: Layout method
	(4) *Spc*: Spacing of drainage facility
	(5) *D*: Depth of drainage facilities
	(6) *V*: Magnitude of vacuum loading
	(7) *SL*: Magnitude of surcharge loading
	(8) *T*: Final measured time of settlement
	(9) $\overline{E}_{sj}$: Compression modulus of *j*th segment
	(10) $\overline{\varphi }_{j}$: Internal friction angle of *j*th segment
	(11) $\overline{e}_{{_{j} }}$: Porosity ratio of *j*th segment
Out parameter	*S*: Settlement

### Model feature selection

In the early stage of database construction, 20 input variables as shown in Table 2 were selected based on the calculation theory of settlement and practical knowledge to guarantee the training quality of the model. However, it is important to note that when dealing with machine learning models, an excessive number of input features does not necessarily improve the fitting accuracy of the model. On the contrary, an overabundance of input features can lead to increased complexity of the model, potentially impacting its processing speed and efficiency. To address this, it is essential to employ data filtering techniques that prioritize selecting input features with a strong correlation to the target variable. This approach not only enhances the training effectiveness of the model but also improves its operational efficiency.

**Figure 1:**
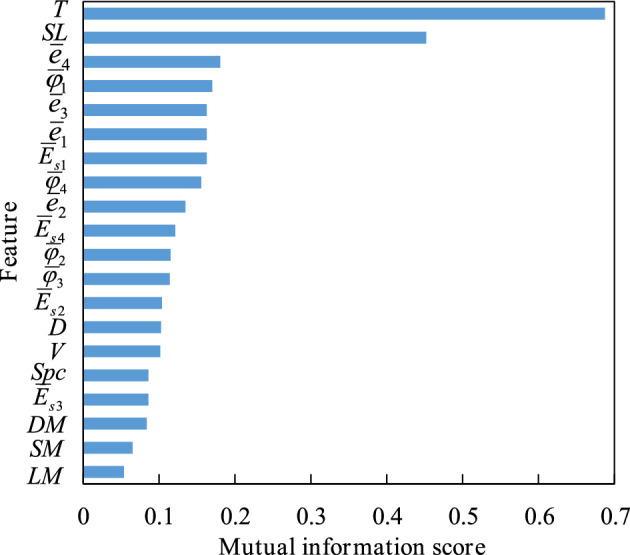
Sorting by mutual information score.

The mutual information method^49^ is an effective feature selection technique that can capture the complex relationships between features and labels. This method can measure the mutual dependence or correlation between features and the target variable, providing a quantitative measure of their relevance. A mutual information score of 0 indicates that the two variables are independent of each other, while a score of 1 indicates that the two variables are entirely related. Figure 1 displays the mutual information scores of all features in the database with respect to settlement, sorted in descending order.

As displayed in Fig. 1, all the features in the database have a mutual information score greater than 0, illustrating that all features in the database have some degree of correlation with the target variable of settlement and the selection of database features was reasonable. To ensure the efficiency and accuracy of the machine learning model, features with a mutual information score greater than 0.1 were chosen as input parameters for the settlement prediction model, and the final input and output parameters for the model are shown in Table 3.

**Table 3:** Model parameter selection after feature analysis.

Classification	Model parameters
Input parameters	$T,SL,\overline{e}_{4} ,\overline{\varphi }_{1} ,\overline{e}_{3} ,\overline{e}_{1} ,\overline{E}_{s1} ,\overline{\varphi }_{4} ,\overline{e}_{2} ,\overline{E}_{s4} ,\overline{\varphi }_{2} ,\overline{\varphi }_{3} ,\overline{E}_{s2} ,D,V$
Out parameters	S

## Theoretical basis and algorithm principles of ISSA-RF model

### Multi-strategy improved sparrow search algorithm

**Figure 2:**
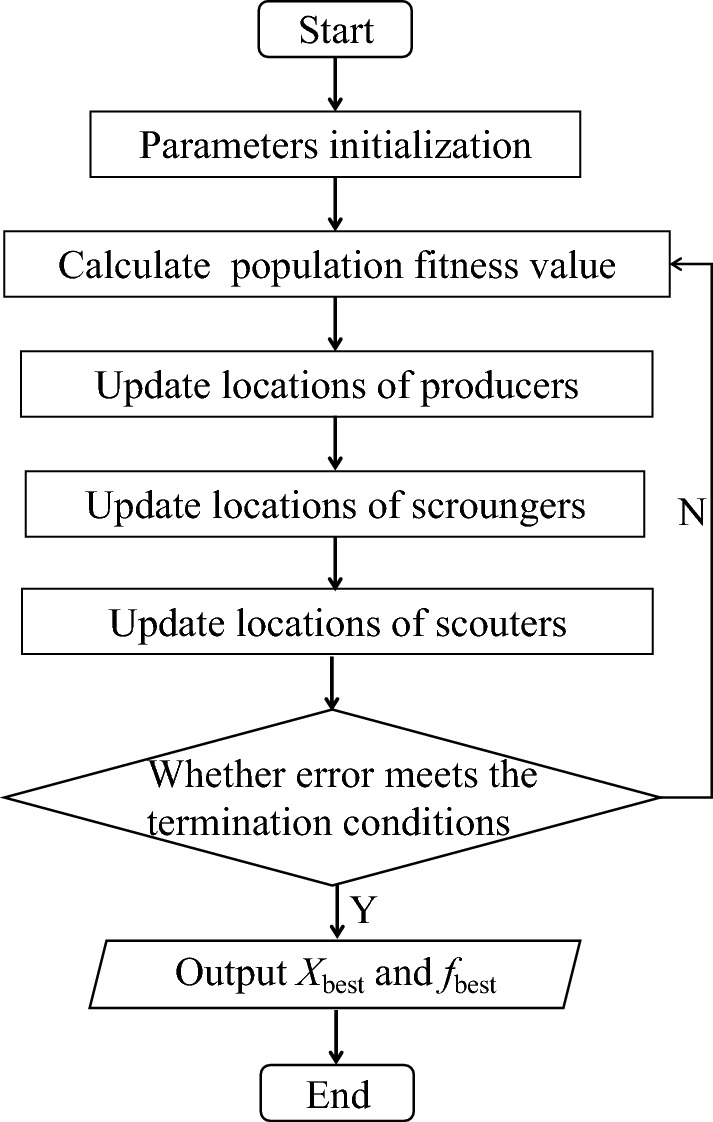
Flowchart of sparrow search algorithm.

The Sparrow Search Algorithm (SSA) is an intelligent optimization algorithm proposed by Xue^50^, and the operational process of the SSA algorithm is depicted in Fig. 2.

Compared to traditional intelligent optimization algorithms, e.g., particle swarm optimization (PSO)^51^ and butterfly optimization algorithm (BOA)^52^, the SSA algorithm has demonstrated significant improvements in convergence speed and accuracy, making it widely applicable in multidisciplinary fields such as traffic flow combination prediction and power prediction. Nevertheless, during the optimization process of the SSA algorithm, issues of insufficient population diversity, slow convergence speed, and susceptibility to local optimization have arisen. To address these issues, the SSA algorithm has been enhanced by improving the population initialization, sparrow finder position updates and global search.

(1) *Population initialization using logistic-tent chaos mapping*. Population initialization is a crucial step in any intelligent optimization algorithm, and the number and dimension of the population directly impact the optimization efficiency of the algorithm. The population initialization of the SSA algorithm relies on randomly generating individuals, which often results in an uneven distribution of the population. This lack of uniformity can significantly hinder the optimization efficiency of the algorithm.

To enhance the optimization efficiency of the algorithm, the SSA algorithm incorporates the Logistic-Tent Chaos Mapping^53^ during the population initialization stage. The Logistic-Tent chaotic mapping is characterized by the following equation:

4 $$x_{n + 1} = \left\{ {\begin{array}{*{20}c} {\left| {rx_{n} (1 - x_{n} ) + \frac{(4 - r)}{2}x_{n} } \right|,x_{n} < 0.5} \\ {\left| {rx_{n} (1 - x_{n} ) + \frac{{(4 - r)(1 - x_{n} )}}{2}x_{n} } \right|,x_{n} \ge 0.5} \\ \end{array} } \right.$$

where *r* is a control parameter; and *x* is a system variable.

(2) *Update finder positions using inertia weight parameter*. To enhance the optimization rate, the SSA algorithm increases the search step size during the update of discoverer positions. However, this increase in step size may result in the algorithm falling into a local optimal state, preventing it from discovering the global optimal solution. To address this issue, an adaptive nonlinear inertia decreasing weight parameter is introduced^14^ during the position update of finders in the SSA algorithm. This effectively prevents finders from converging to local optima while enhancing the global search capability of the algorithm. The expression for the adaptive nonlinear inertia decreasing weights is shown in Eq. (5):

5 $$\omega = \omega_{1} \left( {\omega_{1} - \omega_{2} } \right)\left( {1 - \tan \frac{\pi t}{{4iter_{\max } }}\frac{{t^{2} }}{{iter_{\max }^{2} }}} \right)$$

where $\omega_{1}$ and $\omega_{2}$ are inertia adjustment parameters with values 0.9 and 0.4, respectively; $iter_{\max }$ represents the maximum number of iterations.

(3) *Global optimization with Levy flight strategy*. Upon completing an iteration, the SSA algorithm incorporates the Levy Flight Strategy^54^ to globally perturb the algorithm, enhancing its global search ability and preventing it from getting trapped in local optima. The Levy flight calculation formula is represented as follows in Eq. (6):

6 $${\text{Levy}}(x) = \frac{u}{{\left| v \right|^{{{1 \mathord{\left/ {\vphantom {1 \beta }} \right. \kern-0pt} \beta }}} }}$$

where *u, v* are random numbers that follow a normal distribution, and $\beta \in (0,2)$ are generally taken to be 1.5.

(4) *Performance evaluation of improved sparrow search algorithms*. To verify the performance of the improved sparrow search algorithm (ISSA) in terms of optimization and robustness, its optimization results are compared with those obtained by other algorithms, *i.e.*, SSA, BOA and PSO, using the same benchmark function. The benchmark functions used in this study are listed in Table 4.

**Table 4:** Information of benchmark functions.

Benchmark function	Search space	Optimal value
$F_{1} \left( x \right) = \sum\limits_{i = 1}^{n} {x_{i}^{2} }$	[− 100, 100]	0
$F_{2} \left( x \right) = \sum\limits_{i = 1}^{n} {\left| {x{}_{i}} \right|} + \prod\limits_{i = 1}^{n} {\left| {x{}_{i}} \right|}$	[− 10, 10]	0
$F_{3} \left( x \right) = \sum\limits_{i = 1}^{n} {\left( {\sum\limits_{j = 1}^{i} {x_{j} } } \right)}^{2}$	[− 100, 100]	0
$F_{4} \left( x \right) = \max_{i} \left\{ {\left| {x_{i} } \right|} \right.,1 \le i \le \left. n \right\}$	[− 100, 100]	0
$F_{5} \left( x \right) = \sum\limits_{i = 1}^{n} {\left[ {x_{i}^{2} - 10\cos \left( {2\pi x_{i} } \right) + 10} \right]}$	[− 5.12, 5.12]	0
$F_{6} \left( x \right) = \frac{1}{4000}\sum\limits_{i = 1}^{n} {x_{i}^{2} } + \prod\limits_{i = 1}^{n} {\cos \left( {\frac{{x_{i} }}{\sqrt i }} \right)} + 1$	[− 600, 600]	0

Benchmark functions *F*_1_*–F*_4_ are unimodal, containing only one extreme point within the search space, designed to evaluate the optimization ability of the algorithm. On the other hand, benchmark functions *F*_5_ and *F*_6_ are multimodal, containing multiple extreme points within the search space, intended to assess the ability to escape local optima and discover global optimal solutions.

In accordance with the principles of fairness and justice, the population sizes of the intelligent optimization algorithm are set to 30, and the maximum number of iterations is limited to 500. Additionally, to mitigate the potential impact of errors from a single run and to enhance the credibility of the testing results, the intelligent optimization algorithm is configured to execute independently 30 times for each of the six benchmark functions.

The average value serves as a reliable indicator of the convergence accuracy of the algorithm. Table 5 compares the average values among the four intelligent optimization algorithms for the benchmark function. As indicated in Table 5, under the same conditions, the ISSA algorithm exhibits significantly higher convergence accuracy on both unimodal and multimodal functions compared to the other three algorithms, by orders of magnitude. Moreover, the ISSA algorithm accurately identifies the theoretical optimal solutions for the unimodal functions *F*_1_ and *F*_3_, as well as the multimodal functions *F*_5_ and *F*_6_. Notably, the optimal solutions found for *F*_2_ and *F*_4_ are extremely close to the theoretical optimal solution. These results demonstrate the superior performance of the ISSA algorithm in terms of convergence accuracy.

**Table 5:** Comparison of average values for different benchmark functions.

	ISSA	SSA	BOA	PSO
*F*_1_	0	7.70 × 10^−10^	5.98 × 10^−10^	3.29
*F*_2_	1.88 × 10^−282^	1.28 × 10^−4^	1.28 × 10^−6^	30.22
*F*_3_	0	1.75 × 10^−8^	5.62 × 10^−10^	179.10
*F*_4_	6.68 × 10^−194^	6.83 × 10^−7^	2.17 × 10^−7^	3.75
*F*_5_	0	2.01 × 10^−8^	117.99	160.32
*F*_6_	0	3.92 × 10^−12^	1.16 × 10^−9^	0.37

The standard deviation can reflect the stability and robustness of the algorithm, and Table 6 presents a comparative analysis of the standard deviation among the four intelligent optimization algorithms on the benchmark function. As indicated in the table, the ISSA algorithm exhibits a standard deviation of 0 across various test functions, indicating that the algorithm consistently achieves the optimal value in each iteration. This finding highlights the superior stability of the ISSA algorithm compared to the PSO algorithm, BOA algorithm, and SSA algorithm.

**Table 6:** Comparison of standard deviation for different benchmark functions.

	ISSA	SSA	BOA	PSO
*F*_1_	0	3.01 × 10^−9^	3.62 × 10^−11^	0.84
*F*_2_	0	3.95 × 10^−4^	4.32 × 10^−7^	23.01
*F*_3_	0	8.37 × 10^−8^	4.54 × 10^−11^	58.18
*F*_4_	0	1.73 × 10^−6^	1.05 × 10^−8^	1.23
*F*_5_	0	6.34 × 10^−8^	84.66	25.30
*F*_6_	0	2.03 × 10^−11^	2.25 × 10^−10^	0.09

### Construction of ISSA-RF model

**Figure 3:**
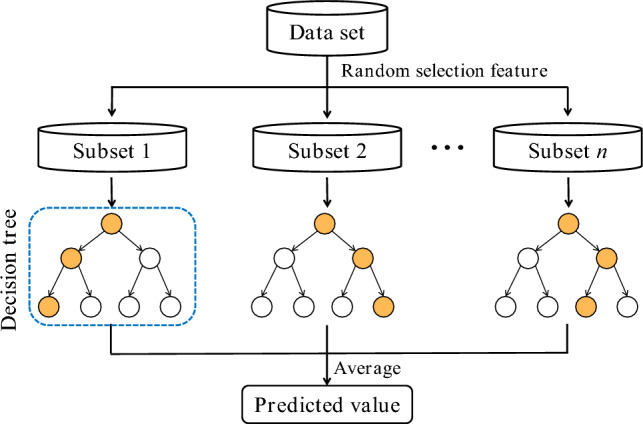
Learning process of random forest model.

The random forest (RF)^52^ model is a widely utilized tree ensemble algorithm that employs multiple decision trees to make predictions, and obtain the final prediction result through various operation modes, such as voting or averaging. In comparison to a single decision tree, random forests have better robustness and prediction accuracy, particularly for small database samples. The learning process of the RF model is depicted in Fig. 3.

The random forest model comprises several hyperparameters that impact the performance of the model, such as the number of decision trees (n_estimators), the maximum depth of each decision tree (max_depth), the minimum number of samples required to split a node in the decision tree (min_samples_split) and the minimum number of samples that a leaf node must contain (min_samples_leaf), and so on. The selection and tuning of these hyperparameters are crucial for optimizing the efficiency and effectiveness of the random forest model. In this study, the n_estimators and max_depth of RF model are suggested to be optimized using the Improved Sparrow Search Algorithm (ISSA). As a result, the construction process of the ISSA-RF model for predicting the settlement of soft foundation is outlined as follows:

1. *Determine the initial parameters of the model*. Set the optimization interval for the hyperparameters to be optimized, and determine the initial parameters of the ISSA algorithm.
2. *Initialize population*. A more uniformly distributed initialized population is generated according to the logistic-tent chaotic mapping. The positions of the optimal, suboptimal, and worst fitness individuals of the RF model and their corresponding fitness values are then recorded.
3. *Update the discoverer location*. Update the position of the individual finder, and calculate its fitness value. If the fitness value of the updated individual is superior, replace the pre-update individual with the updated one; otherwise, there will be no change.
4. *Disturb the population*. Use Levy flight to mutate the population. If the fitness value of the individual after mutation is superior, replace the pre-mutation individual with the mutated one; otherwise, it remains unchanged.
5. *Determine whether the ISSA algorithm is terminated*. If the maximum number of iterations is reached, the ISSA algorithm is terminated. The global optimal solution is output, and the optimized hyperparameters are passed back to the RF prediction model. Otherwise, go to step (3) to recalculate until the stopping condition is met.
6. *Predict the settlement of soft foundation*. The constructed ISSA-RF model is used to predict the settlement of soft foundation.

**Figure 4:**
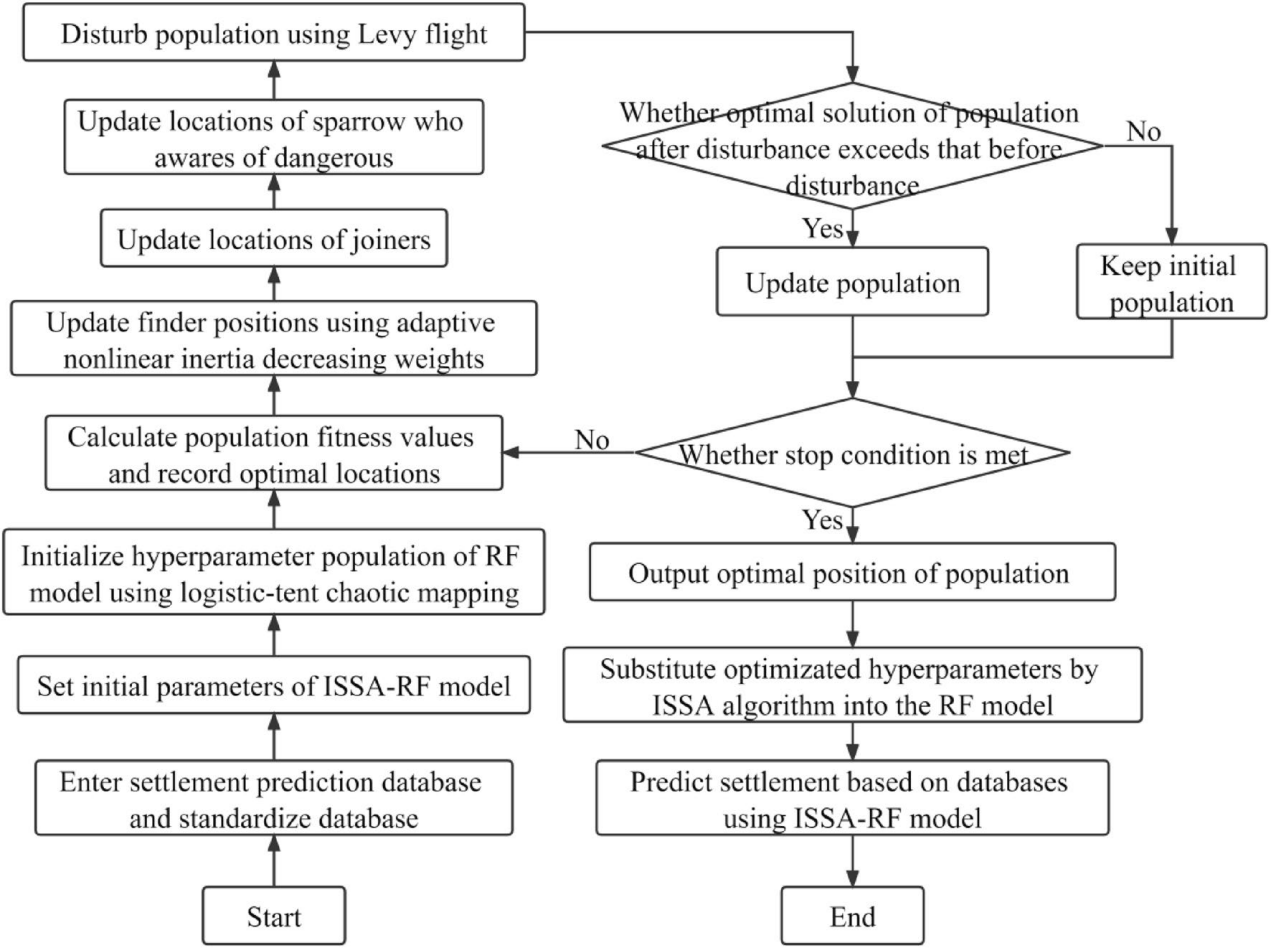
Flowchart of ISSA-RF model predicting settlement.

The flowchart showing the prediction of settlement by the ISSA-RF model is shown in Fig. 4.

## Evaluation of settlement prediction model

### Evaluation indicators

To compare the prediction accuracy of different settlement prediction models, the following four indicators^55–57^ are used in the evaluation:

(1) Root mean square error (RMSE)

7 $${\text{RMSE}} = \sqrt {\frac{1}{n}\sum\limits_{i = 1}^{n} {\left( {y_{i} - \hat{y}_{i} } \right)^{2} } }$$

where *n* is the total number of samples; *i* is the number of sample; $y_{i}$ is the *i*th predicted result of model for settlement; $\hat{y}_{i}$ is the *i*th measured data.

(2) Mean absolute error (MAE)

8 $${\text{MAE}} = \frac{1}{n}\sum\limits_{i = 1}^{n} {\left| {y_{i} - \hat{y}_{i} } \right|}$$

(3) Coefficient of determination (R^2^)

9 $${\text{R}}^{2} = \frac{{(\sum\limits_{i = 1}^{n} {\left({y_{i}-y_{m}) ( \hat{y}_{i}-\hat{y}_{m} )} \right)^{2} } }}{\sum\limits_{i = 1}^{n} {\left({y_{i}-y_{m} } \right)^{2}}{\sum\limits_{i = 1}^{n} {\left({\hat{y}_{i}-\hat{y}_{m} } \right)^{2}}}}$$

where ${y}_{m}$ and $\hat{y}_{m}$ are the average of the predicted and measured values, respectively.

(4) Variance account factor (VAF)

10 $${\text{VAF}} = 1 - \frac{{{\text{var}} \left( {\hat{y}_{i} - y_{i} } \right)}}{{{\text{var}} \hat{y}_{i} }}$$

### Predictions by RF model

**Figure 5:**
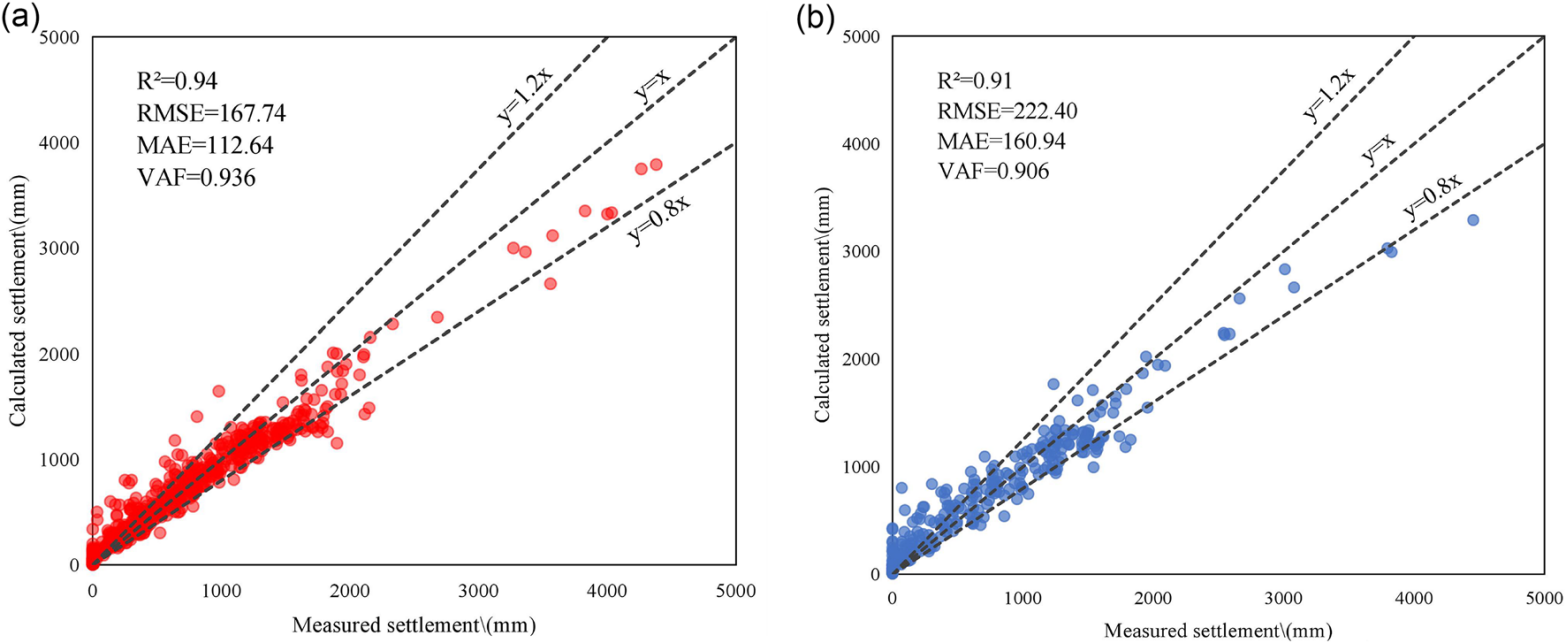
Prediction results of RF model: (**a**) Training set and (**b**) Testing set.

The settlement prediction database constructed in the previous section is divided into a training set and a testing set with a ratio of 8:2. The hyperparameters n_estimators and max_depth in the RF model are set to 100 and 5, respectively. The RF model is trained on the training set and tested on the testing set to verify its prediction ability. The performance of the RF model on the training and testing sets is shown in Fig. 5.

In the training set, the RF model exhibits a high degree of fit with an R^2^ value of 0.94. The distribution plot reveals that when the settlement value is less than 1500 mm, the data is relatively concentrated, and a significant proportion of prediction results fall within the error range of ± 20%. However, due to the fact that the settlement values greater than 1500 mm account for only 10.86% of the settlement prediction database, the learning ability of the model weakens as the settlement value increases beyond 1500 mm. Consequently, the predicted settlement value tends to be lower than the measured value in such cases.

In the testing set, the data distribution is also concentrated when the settlement value is less than 1500 mm, and the predicted value and the measured value are relatively close. However, as the settlement value exceeds 2000 mm, the data distribution becomes more scattered, and the predicted settlement value is less than the measured value. The R^2^ value is only 0.91, and the RMSE, MAE and VAF values are 222.40 mm, 160.94 mm and 0.906, respectively. Compared with the evaluation index of the training set, it is evident that the generalization ability of the RF model is weak.

### Predictions by ISSA-RF model

**Figure 6:**
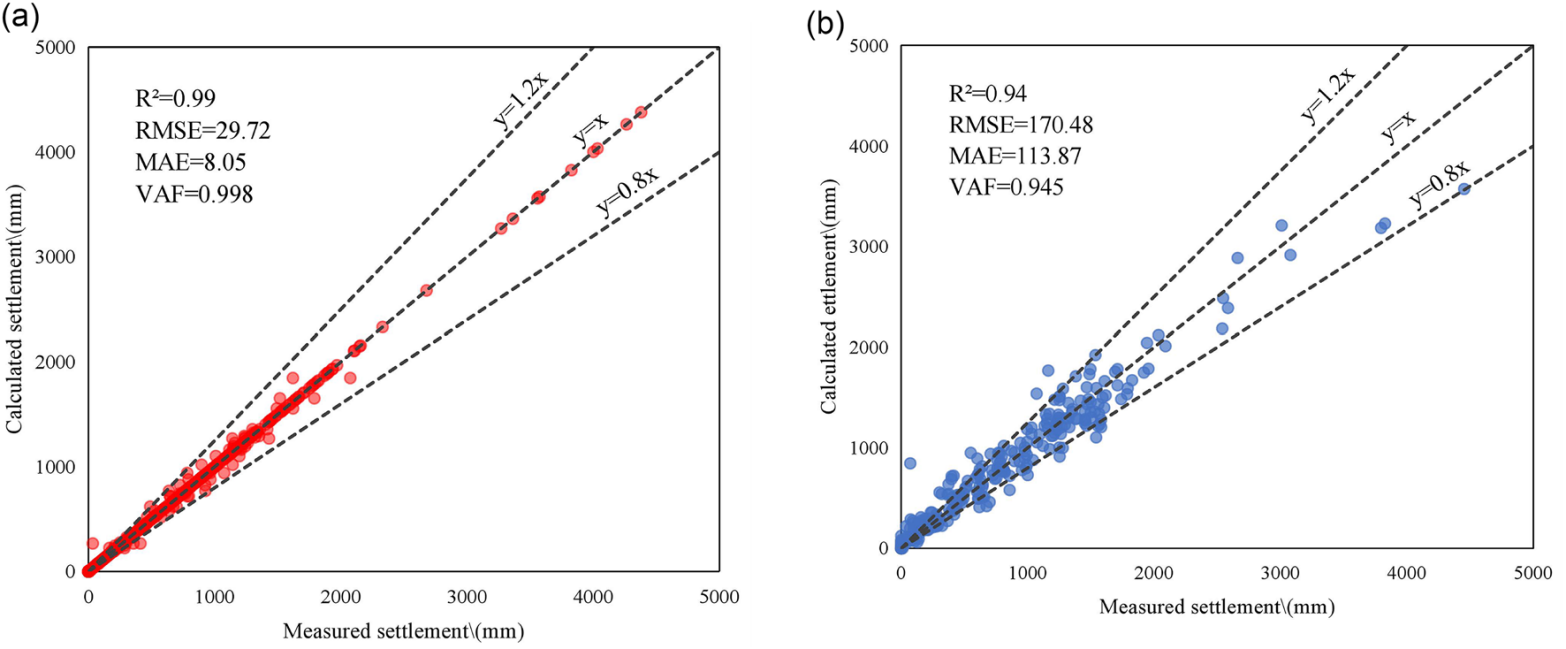
Prediction results of ISSA-RF model: (**a**) Training set and (**b**) Testing set.

The optimization intervals of n_estimators and max_depth are set to [1, 200] and [1. 20], respectively. The sparrow population size, the maximum number of iterations, the dimension and the proportion of finders are set to 50, 300, 2 and 20%, respectively. The optimized values for n_estimators and max_depth are 154 and 17, respectively, and the optimized parameters are transmitted back to the RF model for predicting. The optimal parameters of the RF model and ISSA-RF model are summarized in Table 7. The prediction results for the training and testing sets are presented in Fig. 6.

**Table 7:** Parameters used in making optimal model.

Algorithms used	Hyperparameters		
	Title	Values considered	Optimal
RF	n_estimators	1–200	100
	max_depth	1–20	5
ISSA-RF	n_estimators	1–200	154
	max_depth	1–20	17
	sparrow population size	20–100	50
	maximum number of iterations	100–500	300
	proportion of finders	10–30%	20%

The evaluation indicators in the figure demonstrate that the ISSA algorithm significantly enhances the prediction performance of the RF model on the training and testing sets. The predicted value of the ISSA-RF model on the training set has a high degree of fit with the measured, with the R^2^ value is as high as 0.99, which is 5.32% higher than that of the original RF model. Additionally, the R^2^ value of ISSA-RF model on the testing set is 0.94, which is 3.30% higher than that of the RF model. The RMSE and MAE of ISSA-RF model are 170.48 mm and 113.87 mm, which are smaller than those of the RF model. The VAF values of the ISSA-RF model in both test and training sets are higher than those of the RF model, indicating that the predicted results of the ISSA-RF model are closer to the actual results.

## Applications in project

### Project overview

**Figure 7:**
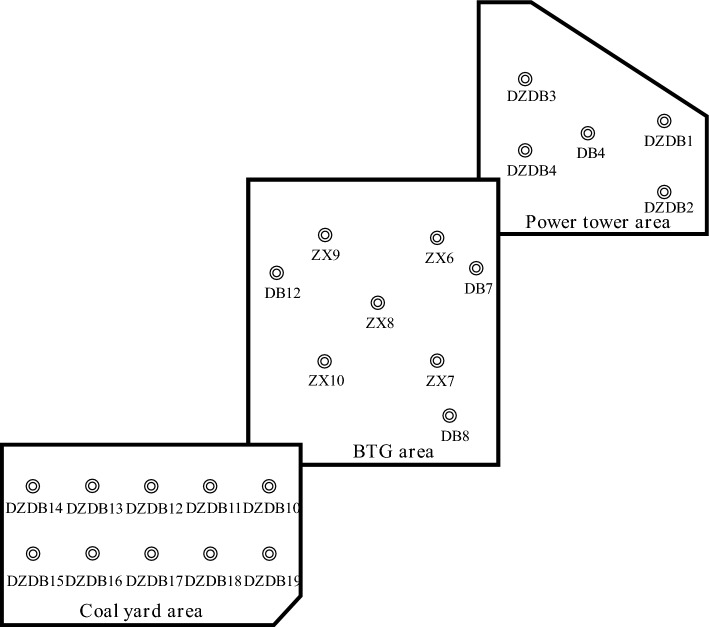
Locations of monitoring points.

The project involves a coal-fired power station situated on an alluvial plain at the river estuary. To facilitate construction, the site has been cleared, raised, and reinforced with surcharge preloading to enhance the bearing capacity of the soft foundation. The surcharge was carried out in three distinct areas, *i.e.* BTG area, power tower area and coal yard area, and the magnitude of the preloading in each area is determined according to the design load of the structure. The size and total preloading values for each area are displayed in Table 8. To effectively monitor settlement and identify the optimal time for unloading, the settlement monitoring points have been set up on the site, and their locations are displayed in Fig. 7.

**Table 8:** Information of surcharge areas.

Surcharge area	Size (*W* × *L*, m)	Magnitude of preloading (kPa)
BTG	270 × 360	112
Power tower	310 × 331	64
Coal yard	333 × 580	160

The surface settlement is measured by burying settlement plates at the monitoring points and using the method of digital level. Settlement is monitored every 5 days during loading phase and once every 7 days during full load phase. If a critical state or abnormal condition occurs, the number of monitoring is increased, and the frequency of monitoring is adjusted appropriately according to the site situation.

### Verification

**Figure 8:**
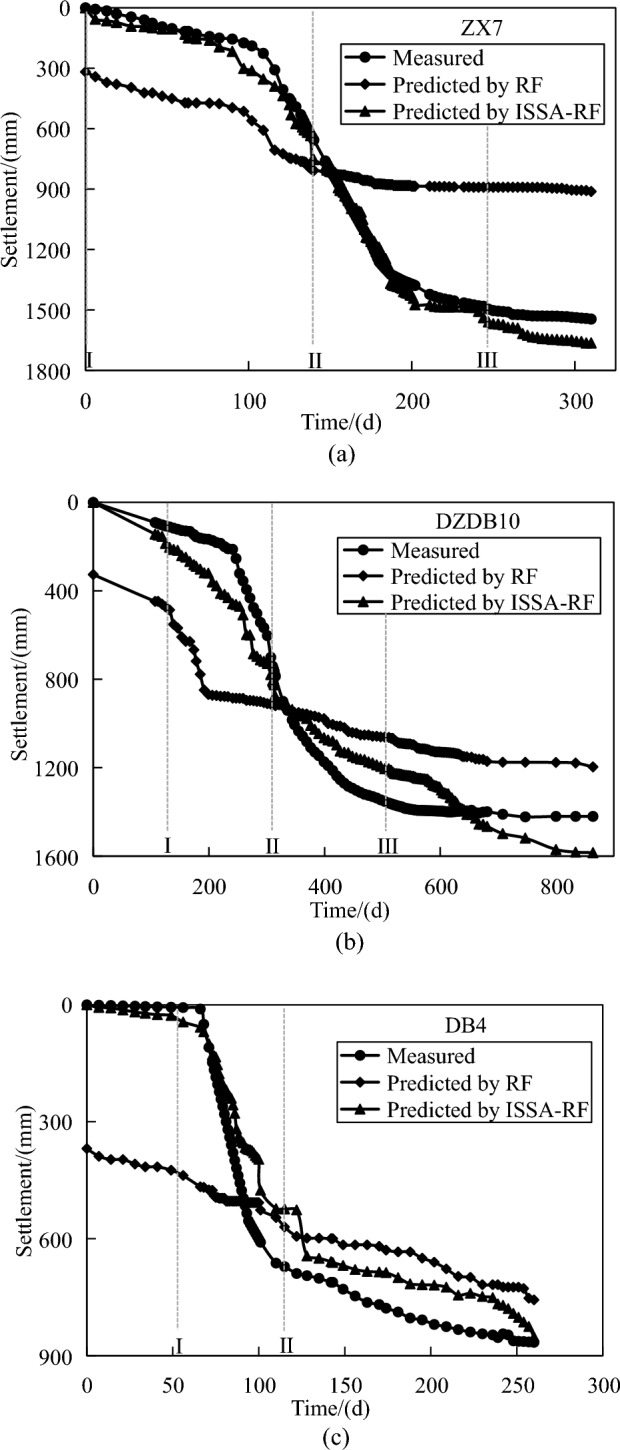
Comparison of the predicted settlements by RF and ISSA-RF with the measured for (**a**) ZX7, (**b**) DZDB10 and (**c**) DB4.

Based on the monitoring data of the surface settlement, it can be obtained that, excluding the influence of adjacent surcharge sites, the settlement development trend in various areas is generally similar. Therefore, only three monitoring points in distinct preloading areas were analyzed for analysis, *i.e.* ZX7, DZDB10 and DB4. To assess the practical application of the ISSA-RF model, the settlement at these three monitoring points was predicted. The predicted results were then compared with those obtained from the RF model, and the comparisons are displayed in Fig. 8 and Table 9.

**Table 9:** Comparison of predictions by two models.

		ZX7	DZDB10	DB4
RF	R^2^	0.28	0.53	0.47
	RMSE/mm	419.51	342.44	217.7
	MAE/mm	374.75	308.59	180.33
	VAF	0.275	0.529	0.470
ISSA-RF	R^2^	0.98	0.93	0.88
	RMSE/mm	60.55	127.69	104.98
	MAE/mm	49.58	116.38	89.81
	VAF	0.985	0.935	0.877

I, II and III in Fig. 8 represent the time to start loading, the time to fully load, and the time to start unloading, respectively. It should be noted that the time nodes for the loading–unloading construction differ among the three sites. Time 0 in ZX7, DZDB10 and DB4 corresponds to March 6, June 18, and June 15, 2020, respectively. By comparing Table 9 and Fig. 8, it can be observed that the prediction results of the RF model at all three points exhibit significant fitting error when compared to the measured data. Moreover, the settlement development reaches approximately 300 mm at the initial monitoring time, which deviates from the expected settlement development pattern. Compared with the excessive prediction error of the RF model, the ISSA-RF model shows strong superiority in the prediction results of three points, particularly during the loading stage from I to II. Its coefficients of determination reach 0.98, 0.93 and 0.88 for ZX7, DZDB10, and DB4, respectively. However, both models exhibit a significant error when predicting the settlement of DB4 points. This could be attributed to the overload preload and high surcharge rate prevalent in this area, leading to a rapid settlement after reaching full load, a phenomenon that the models have not been able to capture effectively.

Comparing the settlement prediction results of the RF model and the ISSA-RF model, it can be seen that the values of the model hyperparameters (n-estimators and max-depth) have a significant impact on the prediction effect of the model. After optimizing the model hyperparameters by using the sparrow search algorithm improved by logistic-tent chaotic mapping, adaptive nonlinear decreasing inertia weight parameters and levy flight strategy, the efficiency of RF model in learning the settlement law of soft ground can be significantly improved. In addition, Table 10 provides a comparative analysis of the performance of the proposed model with other methods in predicting the maximum settlement value. The proposed method exhibits clear advantages over other algorithms, and the prediction results obtained in this study are relatively consistent and stable.

**Table 10:** Comparison of predicted results of maximum settlement.

	Measured maximum settlement/mm	Predicted maximum settlement/mm and relative error (%)				
		ISSA-RF	RF	Asaoka	Exponential curve method	Hyperbolic method
ZX7	1546.56 /	1664.27 7.61%	911.98 41.03%	1923.49 24.37%	781.82 49.45%	1899.31 22.81%
DZDB10	1420.43 /	1584.44 11.55%	1196.64 15.76	1445.63 1.77%	371.48 73.85%	1545.82 8.83%
DB4	865.83 /	851.74 1.63%	757.03 12.57%	763.54 11.81%	587.91 32.10%	1424.56 64.53%

It is worth noting that the ISSA-RF model can be utilized in various soft foundation treatment projects. Furthermore, based the construction plan and survey report, the model can effectively predict the treatment effect prior to commencing construction. This provides valuable guidance and serves as a reference for the actual construction process. However, the algorithm has high professional requirements and can be challenging for engineers to apply. To enhance its accessibility, it is recommended that the relevant algorithms be developed into software for widespread promotion and application.

## Conclusions

Based on a large number of data from the preloading projects in the soft ground, this paper establishes a settlement database, and constructs a settlement prediction model using the random forest model optimized by improved sparrow search algorithm. The following concluding remarks have been deduced:

1. The optimization ability and accuracy of the sparrow search algorithm (SSA) can be increased through the implementation of an improved sparrow search algorithm (ISSA) that incorporates the logistic-tent chaos mapping, adaptive nonlinear decreasing inertia weight parameter, and Levy flight strategy.
2. When the ISSA algorithm was applied to the random forest model, the resulting ISSA-RF model exhibited a 13.41% improvement in performance compared to the RF model.
3. In practical applications, the ISSA-RF settlement prediction model outperforms the RF model in predicting settlement throughout the entire loading–unloading process, as well as the maximum settlement. It exhibits greater practicality and can be effectively utilized in real-world engineering projects to provide valuable insights for formulating construction plans.

It is important to note that the settlement of a site is influenced by the loading of adjacent sites. However, the model lacks the capability to account for this interaction, which remains a challenge to be addressed in future iterations.

## Data Availability

The datasets used and/or analysed during the current study are available from the corresponding author on reasonable request.
